# Development and validation of machine learning algorithms based on electrocardiograms for cardiovascular diagnoses at the population level

**DOI:** 10.1038/s41746-024-01130-8

**Published:** 2024-05-18

**Authors:** Sunil Vasu Kalmady, Amir Salimi, Weijie Sun, Nariman Sepehrvand, Yousef Nademi, Kevin Bainey, Justin Ezekowitz, Abram Hindle, Finlay McAlister, Russel Greiner, Roopinder Sandhu, Padma Kaul

**Affiliations:** 1 Department of Computing Science, University of Alberta, Edmonton, AB Canada; 2 Canadian VIGOUR Centre, Department of Medicine, University of Alberta, Edmonton, AB Canada; 3 Department of Medicine, University of Alberta, Edmonton, AB Canada; 4 Department of Medicine, University of Calgary, Calgary, AB Canada; 5 Smidt Heart Institute, Cedars-Sinai Medical Center Hospital System, Los Angeles, CA USA

**Keywords:** Diagnosis, Cardiovascular diseases

## Abstract

Artificial intelligence-enabled electrocardiogram (ECG) algorithms are gaining prominence for the early detection of cardiovascular (CV) conditions, including those not traditionally associated with conventional ECG measures or expert interpretation. This study develops and validates such models for simultaneous prediction of 15 different common CV diagnoses at the population level. We conducted a retrospective study that included 1,605,268 ECGs of 244,077 adult patients presenting to 84 emergency departments or hospitals, who underwent at least one 12-lead ECG from February 2007 to April 2020 in Alberta, Canada, and considered 15 CV diagnoses, as identified by International Classification of Diseases, 10th revision (ICD-10) codes: atrial fibrillation (AF), supraventricular tachycardia (SVT), ventricular tachycardia (VT), cardiac arrest (CA), atrioventricular block (AVB), unstable angina (UA), ST-elevation myocardial infarction (STEMI), non-STEMI (NSTEMI), pulmonary embolism (PE), hypertrophic cardiomyopathy (HCM), aortic stenosis (AS), mitral valve prolapse (MVP), mitral valve stenosis (MS), pulmonary hypertension (PHTN), and heart failure (HF). We employed ResNet-based deep learning (DL) using ECG tracings and extreme gradient boosting (XGB) using ECG measurements. When evaluated on the first ECGs per episode of 97,631 holdout patients, the DL models had an area under the receiver operating characteristic curve (AUROC) of <80% for 3 CV conditions (PTE, SVT, UA), 80–90% for 8 CV conditions (CA, NSTEMI, VT, MVP, PHTN, AS, AF, HF) and an AUROC > 90% for 4 diagnoses (AVB, HCM, MS, STEMI). DL models outperformed XGB models with about 5% higher AUROC on average. Overall, ECG-based prediction models demonstrated good-to-excellent prediction performance in diagnosing common CV conditions.

## Introduction

The 12-lead electrocardiogram (ECG) is the most common, low-cost, and accessible diagnostic tool for cardiovascular (CV) disease. It is performed on nearly all acute care visits and commonly more than once. In the US alone, over 100 million ECGs are obtained annually^1^. This is useful as the ECG contains a large amount of information that provides insight into underlying cardiac physiology since morphological and temporal features are produced from the electrical activity of the heart. However, standard techniques used by physicians and by computer algorithms to interpret ECGs are constrained, as many are rule-based and only consider a fraction of the total information available on the ECG. Manual or computerized approaches and even conventional statistical methods cannot account for high-level interactions between ECG signals from multiple leads or imperceptible, yet informative, changes that may signal early disease. The emergence of deep learning (DL) analyses offers an exciting opportunity to identify clinically relevant but ‘hidden’ patterns in ECG signals and simultaneously assess complex interactive relationships from routinely captured clinical data for diagnosis of various CV abnormalities^2–5^.

Prior investigations exploring machine-learned models for disease prediction using ECGs have predominantly focused on cardiac conditions that can be readily interpreted by physician experts based on morphological changes in ECG patterns—e.g., arrhythmias (atrial fibrillation [AF], ventricular tachycardia [VT], supraventricular tachycardia [SVT])^6^, ST-elevation myocardial infarction (STEMI) or non-STEMI (NSTEMI)^7–9^, or heart block conditions such as atrioventricular blocks or branch blocks including left-bundle branch block and right-bundle branch block^10^. While the number of machine learning (ML)-based models using ECG data to predict CV conditions beyond those traditionally associated with ECG patterns is currently limited, it is steadily increasing. These models focus on conditions such as mitral valve prolapse (MVP)^1^, cardiac arrest (CA)^11,12^, heart failure (HF)^13,14^, pulmonary embolism (PE)^15,16^, aortic stenosis (AS)^17–19^, mitral valve stenosis (MS)^20^, pulmonary hypertension (PHTN)^21,22^ and hypertrophic cardiomyopathy (HCM)^18,23,24^. Furthermore, while existing studies have mainly concentrated on individual labels, there hasn’t been any prior research developing a predictive system for the simultaneous detection of these specific conditions. The lack of large medical datasets that are clinically annotated with an extensive set of diagnostic labels available for supervised ML is a well-recognized problem, and large-scale validations at the population scale are critical to show trustworthiness for the successful adoption of prediction models into clinical practice, where early identification and treatment may potentially impact disease-related complications, healthcare use, and cost.

Accordingly, we used a large population-level cohort of patients, from a single-payer universal health system, to develop and validate DL models (based on 12-lead ECG tracings) as well as extreme gradient boosting (XGB) models (based on routinely collected ECG measurements) to simultaneously predict 15 common CV diagnoses through a unified prediction framework.

## Results

### Patient characteristics and outcomes

**Fig. 1:**
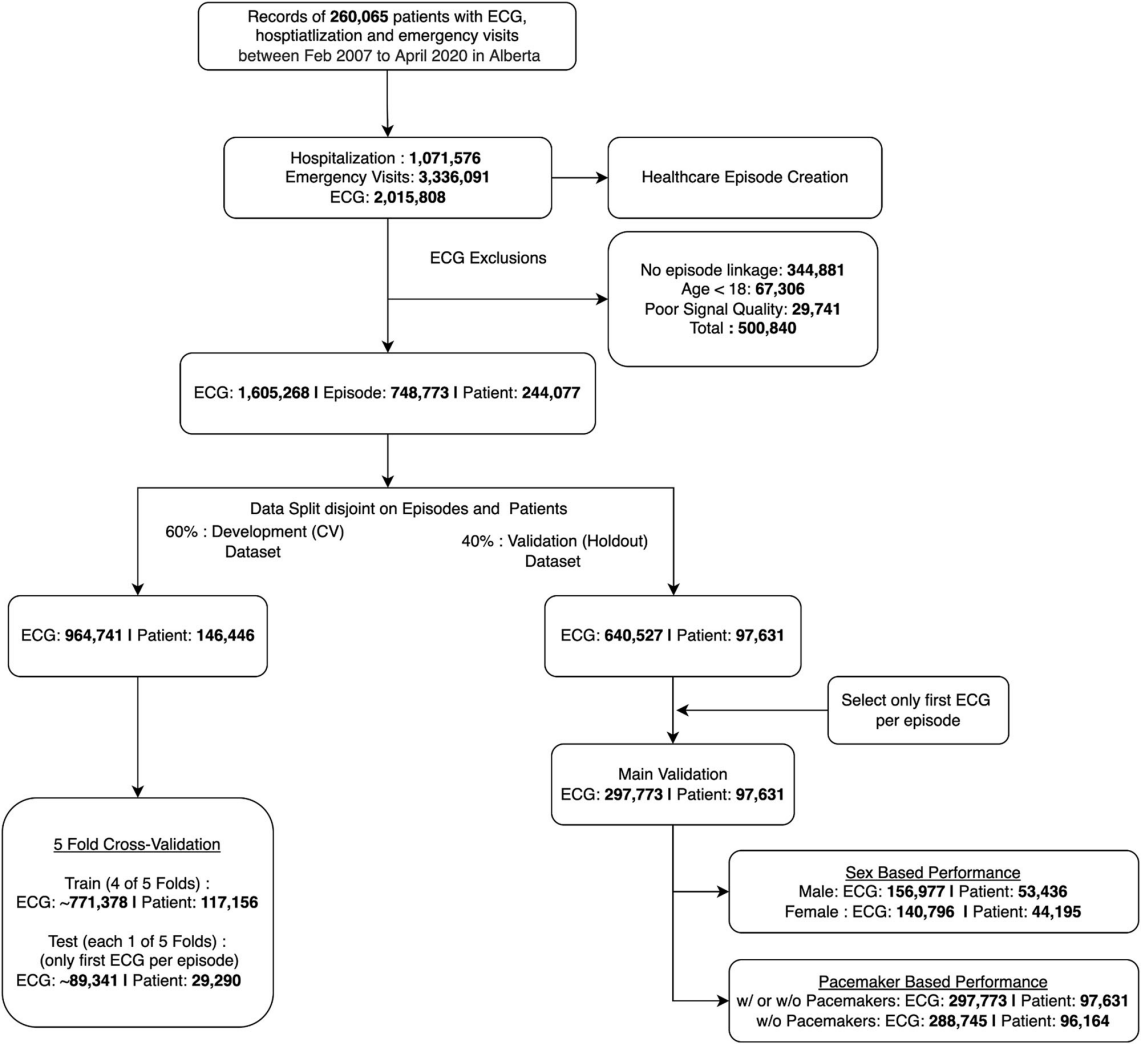
Flowchart of the study design showing the sample sizes for different experimental splits. We divided the entire ECG dataset, allocating 60% for model development (including fivefold internal cross-validation for training and fine-tuning) and setting aside 40% as a holdout set for final validation. For evaluation, we assessed our models using two approaches: first, exclusively on the first ECGs from each episode captured during an ED visit or hospitalization, reflecting the intended point-of-care deployment; second, on all ECGs from the holdout set. Additionally, we evaluated our models’ performance within specific patient subgroups categorized by sex and the presence of cardiac pacing or ventricular assist devices.

Baseline characteristics of the cohort have been described previously^25^. In brief, the average age of patients was 65.8 ± 17.3 years, and 56.7% were males (Supplementary Table 1). The models underwent training using ECGs from 146,446 patients and were subsequently evaluated on a holdout cohort of 97,631 patients (Fig. 1). The holdout dataset included 53,436 men and 44,195 women, used for sex-based performance evaluations. Additionally, 96,164 patients without pacemakers were evaluated separately to investigate the impact of pacemakers on model performance. Anticipating the implementation of our prediction system at the point of care, we assessed our models exclusively using the first ECG of each holdout patient in a specific episode.

Frequency and percentage of ECGs with any of the selected CV conditions in full, development and holdout splits as well as among the first ECG per episode in the holdout set are presented in Table 1. The first ECG per episode in the holdout set (used for the final evaluations) had some differences in diagnostic labels compared to the full ECG data (e.g., frequency for HF: 9.3% vs 15.5%; AF: 11.5% vs 18.2%).

**Table 1:** Frequency and percentage of ECGs with selected cardiovascular conditions in cohorts used in the study

	Full Data (*n* = 1,605,268)	Development set (*n* = 964,741)	Holdout set (*n* = 640,527)	First ECG per episode in holdout set (*n* = 297,773)
Non-ST-elevation myocardial infarction	162,274 (10.11%)	96,828 (10.04%)	65,446 (10.22%)	10,713 (3.60%)
ST-elevation myocardial infarction	100,206 (6.24%)	60,381 (6.26%)	39,825 (6.22%)	5529 (1.86%)
Heart failure	249,325 (15.53%)	150,055 (15.55%)	99,270 (15.50%)	27,820 (9.34%)
Unstable angina	43,466 (2.71%)	26,223 (2.72%)	17,243 (2.69%)	3416 (1.15%)
Atrial fibrillation	302,146 (18.82%)	180,191 (18.68%)	121,955 (19.04%)	34,139 (11.46%)
Ventricular tachycardia	29,672 (1.85%)	17,385 (1.80%)	12,287 (1.92%)	1793 (0.60%)
Cardiac arrest	40,505 (2.52%)	24,100 (2.50%)	16,405 (2.56%)	2844 (0.96%)
Supraventricular tachycardia	24,146 (1.50%)	14,286 (1.48%)	9860 (1.54%)	2222 (0.75%)
Atrioventricular block	40,013 (2.49%)	23,936 (2.48%)	16,077 (2.51%)	2942 (0.99%)
Pulmonary embolism	32,485 (2.02%)	19,458 (2.02%)	13,027 (2.03%)	4763 (1.60%)
Aortic stenosis	30,120 (1.88%)	18,281 (1.89%)	11,839 (1.85%)	3210 (1.08%)
Pulmonary hypertension	36,331 (2.26%)	21,869 (2.27%)	14,462 (2.26%)	4017 (1.35%)
Hypertrophic cardiomyopathy	4485 (0.28%)	2904 (0.30%)	1581 (0.25%)	409 (0.14%)
Mitral valve prolapse	24,481 (1.53%)	14,270 (1.48%)	10,211 (1.59%)	1949 (0.65%)
Mitral valve stenosis	2925 (0.18%)	1743 (0.18%)	1182 (0.18%)	277 (0.09%)

### Model performances and comparison

**Fig. 2:**
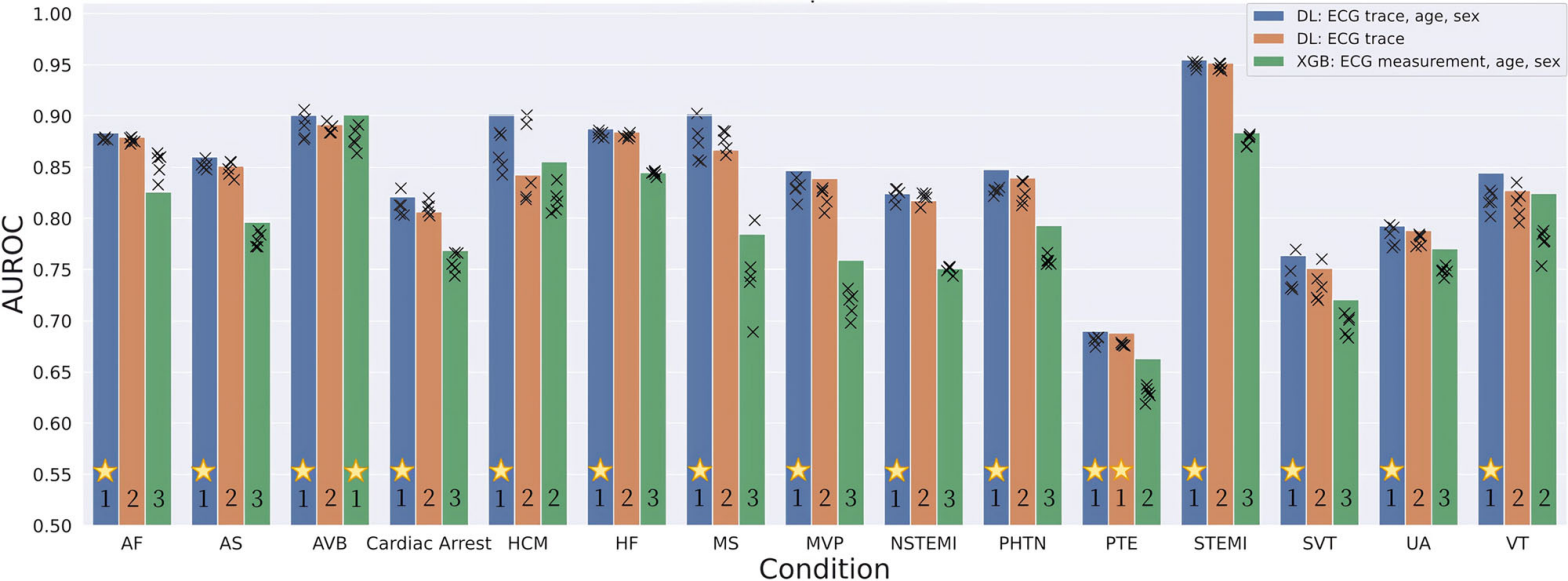
Comparison of AUROC model performances for DL and XGB models with ECG traces (with and without age and sex features) and measurements for 15 cardiovascular conditions. The height of the bars represents the performance in external holdout validation, and the crosses represent the performance in each of the fivefold cross-validation. For each condition, the models are ranked based on their performance (statistically similar performances are assigned tied ranking), and the model with the highest performance is indicated with a star. AF atrial fibrillation, AS aortic stenosis, AUROC Area under the operating receiver curve, AVB atrioventricular block, DL deep learning (ResNet), ECG electrocardiogram, HCM hypertrophic cardiomyopathy, HF heart failure, MS mitral stenosis, MVP mitral valve prolapse, NSTEMI non-ST-elevation myocardial infarction, STEMI ST-elevation myocardial infarction, SVT supraventricular tachycardia, PHTN pulmonary hypertension, PTE pulmonary thromboembolism, UA unstable angina, VT ventricular tachycardia, XGB XGBoost.

Comparison of model performances for DL and XGB models with ECG traces (with versus without age and sex features) and measurements (with age and sex features) for 15 CV conditions is presented in Fig. 2, Table 2 and Supplementary Table 2. The holdout validation of our main model (DL: ECG trace, age, sex) showed that our model for STEMI had the best performance with a receiver operating characteristic curve (AUROC) of 95.5%, and our model for pulmonary thromboembolism (PTE) was the worst performance with an AUROC of 68.9%. The models for all diagnoses, except for PTE, had AUROCs above 76%: with AUROCs <80% for two diagnoses (SVT, UA, in increasing order); AUROCs in the 80–90% range for eight diagnoses in (cardiac arrest [CA], NSTEMI, VT, mitral valve prolapse [MVP], pulmonary hypertension [PHTN], aortic stenosis [AS], AF, HF, in increasing order); AUROCs >90% for four diagnoses (atrioventricular block [AVB], hypertrophic cardiomyopathy [HCM], mitral valve stenosis [MS], STEMI, in increasing order). The model for AF had the highest area under the precision-recall curve (AUPRC) score of 59.2% (F1 score: 51.6%), followed by HF with 56.1% (F1 score: 46.6%) and STEMI with AUPRC of 54.3% (F1 score: 39.2%).

**Table 2:** Evaluation of deep learning: ECG, age, sex model performances for different cardiovascular conditions expressed in mean (95% confidence interval) percentage

	AUROC	AUPRC	F1 Score	Specificity	Recall	Precision	Accuracy	Brier score
Non-ST-elevation myocardial infarction	82.416 (82.412–82.420)	39.509 (39.503–39.514)	21.611 (21.607–21.615)	83.779 (83.778–83.781)	64.781 (64.772–64.791)	12.969 (12.966–12.972)	83.096 (83.095–83.097)	3.477
ST-elevation myocardial infarction	95.486 (95.483–95.489)	54.323 (54.316–54.329)	39.174 (39.166–39.182)	95.471 (95.471–95.472)	82.652 (82.641–82.662)	25.671 (25.665–25.678)	95.233 (95.233–95.234)	1.126
Heart failure	88.758 (88.756–88.760)	56.163 (56.160–56.167)	46.668 (46.664–46.672)	84.376 (84.375–84.378)	76.577 (76.572–76.582)	33.561 (33.557–33.564)	83.648 (83.646–83.649)	6.35
Unstable angina	79.263 (79.256–79.271)	36.583 (36.575–36.591)	5.743 (5.741–5.745)	73.758 (73.756–73.759)	69.826 (69.810–69.841)	2.995 (2.994–2.996)	73.713 (73.711–73.714)	1.233
Atrial fibrillation	88.360 (88.358–88.362)	59.228 (59.225–59.231)	51.555 (51.551–51.559)	84.199 (84.198–84.200)	77.109 (77.105–77.113)	38.723 (38.719–38.726)	83.386 (83.385–83.387)	7.154
Ventricular tachycardia	84.430 (84.420–84.439)	33.790 (33.778–33.801)	6.076 (6.072–6.079)	88.189 (88.188–88.190)	64.175 (64.153–64.197)	3.189 (3.187–3.191)	88.044 (88.043–88.045)	0.627
Cardiac arrest	82.107 (82.099–82.115)	31.270 (31.260–31.280)	8.366 (8.362–8.370)	88.233 (88.232–88.234)	57.625 (57.607–57.643)	4.510 (4.508–4.513)	87.940 (87.939–87.942)	0.942
Supraventricular tachycardia	76.369 (76.358–76.380)	29.031 (29.020–29.042)	4.621 (4.619–4.624)	83.169 (83.167–83.170)	55.317 (55.297–55.338)	2.411 (2.410–2.413)	82.961 (82.960–82.962)	0.683
Atrioventricular block	90.070 (90.064–90.076)	40.544 (40.536–40.553)	11.396 (11.392–11.401)	88.663 (88.662–88.664)	74.669 (74.653–74.685)	6.169 (6.167–6.171)	88.524 (88.523–88.526)	0.874
Pulmonary embolism	68.990 (68.983–68.998)	31.120 (31.113–31.128)	5.572 (5.571–5.574)	68.383 (68.381–68.385)	58.655 (58.641–58.669)	2.925 (2.924–2.926)	68.228 (68.226–68.229)	1.556
Aortic stenosis	85.996 (85.990–86.002)	37.640 (37.632–37.648)	8.084 (8.081–8.087)	82.817 (82.815–82.818)	70.677 (70.661–70.692)	4.287 (4.285–4.289)	82.686 (82.685–82.687)	1.034
Pulmonary hypertension	84.773 (84.768–84.779)	38.091 (38.083–38.098)	9.424 (9.421–9.428)	81.805 (81.804–81.806)	70.738 (70.724–70.752)	5.049 (5.047–5.050)	81.656 (81.654–81.657)	1.296
Hypertrophic cardiomyopathy	90.204 (90.188–90.219)	35.013 (34.990–35.036)	2.471 (2.468–2.474)	92.578 (92.577–92.578)	68.724 (68.679–68.770)	1.258 (1.257–1.260)	92.545 (92.544–92.546)	0.142
Mitral valve prolapse	84.670 (84.661–84.678)	34.646 (34.635–34.656)	5.364 (5.361–5.367)	84.820 (84.818–84.821)	66.275 (66.254–66.296)	2.795 (2.794–2.797)	84.698 (84.697–84.700)	0.645
Mitral valve stenosis	90.228 (90.209–90.247)	39.292 (39.268–39.317)	1.129 (1.127–1.130)	87.289 (87.287–87.290)	77.996 (77.947–78.045)	0.568 (0.568–0.569)	87.280 (87.279–87.281)	0.094

*AUPRC* Area under the precision-recall curve, *AUROC* area under the receiver operating curve, *ECG* electrocardiogram.

The DL model with (ECG trace, age, sex) performed better than the XGB model with (ECG measurements, age, sex) for most diagnoses, except for AVB, where both models performed comparably. DL models outperformed XGB models with an average improvement in AUROC of 5.2%, with notable increases of 11.8% for MS, 8.6% for MVP, 7.3% for NSTEMI, and 7.1% for STEMI. Comparison of 95% confidence intervals from the bootstrap results showed that there were significant differences between DL model performances with versus without (age, sex) features for all diagnoses except PTE, suggesting that age and sex features can add small but significant improvements to diagnostic prediction. Similarly, bootstrap results showed that DL models with ECG traces alone outperformed XGB models with ECG measurements, age, and sex for diagnoses other than AVB, HCM, and VT.

### Sex-based model performance

**Fig. 3:**
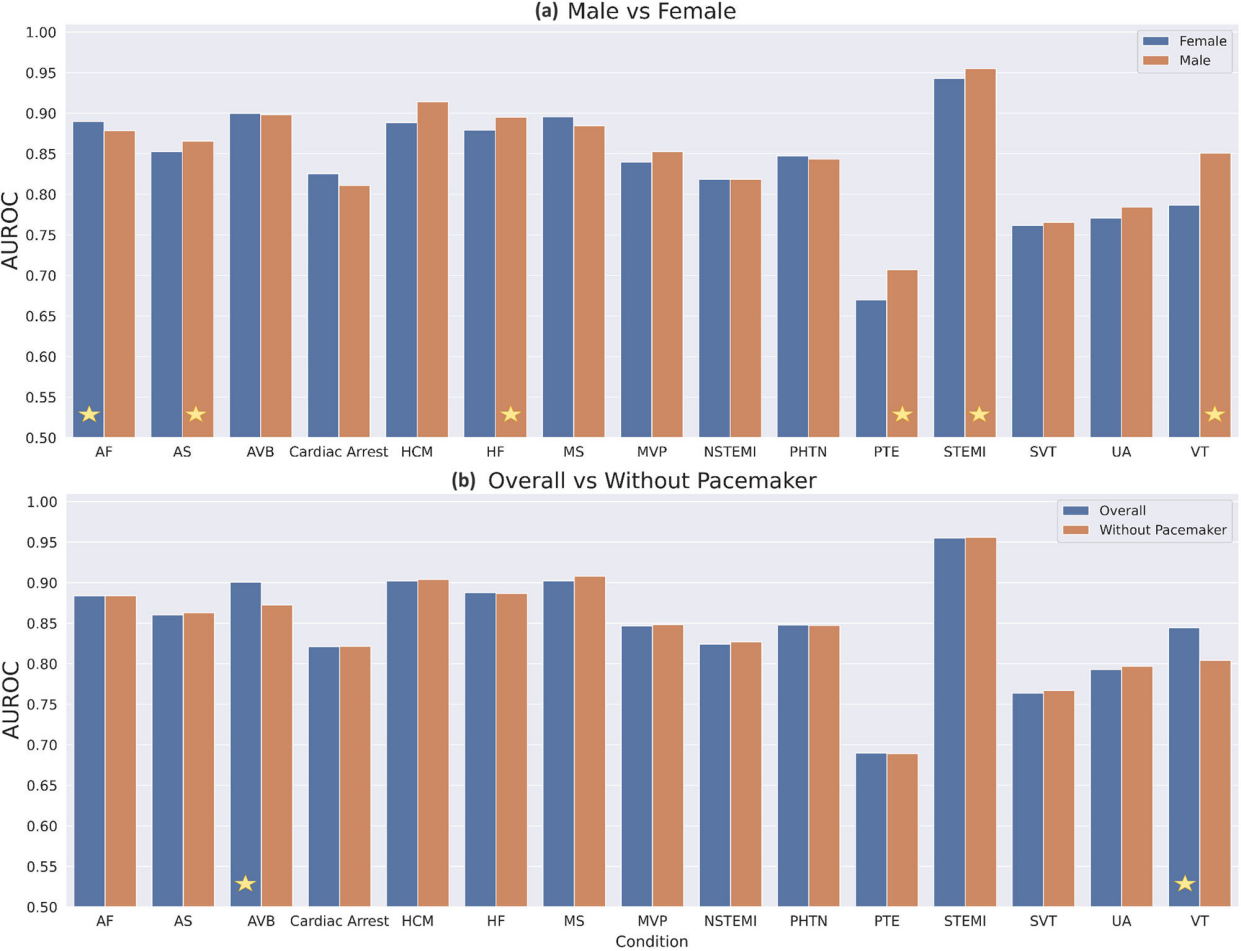
Comparison of AUROC performances for DL: ECG, age, sex model for 15 cardiovascular conditions for specific subgroups. Evaluations are performed separately for the males and females of the holdout patients (**a**) as well as ECGs without pacemakers and all ECGs in the holdout set (**b**). The height of bars represents the performance in external holdout validation and the models with statistically higher performance are indicated with a star.

We evaluated the DL model, which was trained using (ECG trace, age, sex) separately for males and females in the holdout set, and found similar results overall (Fig. 3, top panel). The models performed marginally better for men in 10 of out 15 conditions, with average AUROC increase of 1.0%. Five of these—namely VT, STEMI, PTE, HF, and AS—showed significant differences. We found the highest difference in VT where the model performed 6.4% better for men compared to women (Men: 85.1%, Women: 78.7%), and 14.8% in terms of AUPRC (Men: 37.5%, Women: 22.6%). In contrast, prediction performance for AF was significantly better by 1.2% AUROC in females than in males.

### Pacemaker presence-based model performance

Similarly, evaluating the DL (ECG trace, age, sex) models on the holdout ECGs, after excluding the ECGs of patients with pacemakers and other ICD devices, showed performance that is comparable with overall evaluation, with a very small average AUROC increase of 0.25% with vs without those ECGs (Fig. 3, bottom panel). Again, VT showed the highest difference, where performance dropped by 3.2% AUROC and 5.6% AUPRC when pacemaker ECGs were excluded. Another diagnosis that showed significant difference in the same direction was AVB (1.6% AUROC drop, 5.7% decrease in AUPRC).

### Leave-one-hospital-out validation

Our study utilized ECGs sourced from 14 hospitals. Notably, two of these were tertiary care hospitals, contributing the highest ECG counts (487,042 and 453,085 ECGs, respectively). For each tertiary hospital (H1 and H2), we performed a leave-one-hospital-out validation, training on ECGs from all other hospitals, excluding the validation one (Supplementary Figure 1). The performance of the DL: ECG, Age, Sex model with leave-one-hospital-out validation was comparable to the results reported on the overall holdout set (Supplementary Table 3). In comparison to the primary validation outcomes, the average AUROC performance over 15 conditions exhibited a slight increase of 1.38% in H1 validation but a decrease of 1.34% in H2 validation.

### All-ECG holdout evaluation

As an added validation of our DL model, we evaluated its performance in all ECGs, rather than just the first ECG, acquired during each episode of care for patients in the holdout set. The results, as outlined in Supplementary Table 4, exhibit performance that is either superior or comparable to that achieved using only the initial ECGs.

The all-ECG evaluation revealed some fluctuations in AUROC and AUPRC scores. There was an overall decrease of 2.04% in the average AUROC (averaged across 15 conditions) and a concurrent increase of 2.15% in AUPRC for the all-ECG assessment. Notably, F1-scores for all labels in the all-ECG evaluation exhibited improvements ranging from 1.09% to 16.81%, with an average increase of 6.28%. Similarly, positive predictive values (PPV or precision) for all labels showed an increase ranging from 0.56% to 15.32%, with an average improvement of 4.98%. Therefore, these algorithms can be anticipated to exhibit comparable, if not superior, performance when applied to ECGs conducted at any point during the course of an episode of care.

### Composite label evaluation

The prevalence of several of the diagnoses of interest in our sample was low (e.g., 0.09% for MS among first ECGs), which is likely to impact PPV. We, therefore, explored an alternative evaluation scheme based on a composite label approach that has been previously employed for screening purposes to enhance diagnostic yield^20^. We created a composite label such that it was positive if any of our 15 conditions of interest were positive, and negative if all of the conditions were negative.

We re-evaluated our multi-label DL model’s ability to predict if an ECG is positive for the composite label. Results showed PPV of 31.64% with a F1 score of 47.39%. We also trained a new model supervised with the composite label using the same model architecture and assessed its performance on the same holdout set. Results showed PPV of 57.9% with a F1 score of 63.03%. These results suggest that a higher PPV could be achieved when screening for the composite outcome.

### Model explanations

**Fig. 4:**
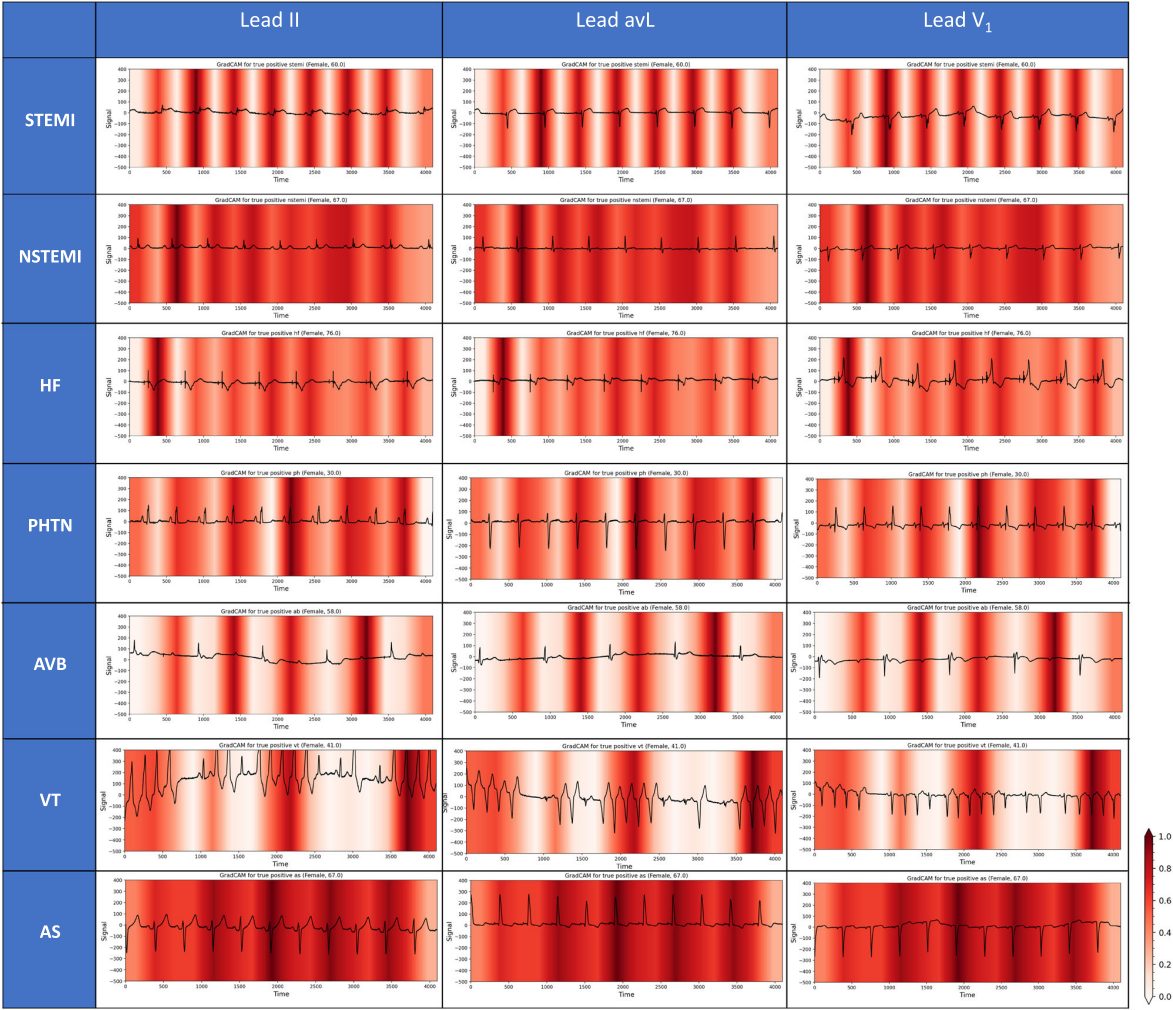
The GradCAM plots for the DL model in diagnosis of different cardiovascular conditions. Representative ECG traces were chosen for a selected group of diagnoses. GradCAM results do not extend to the entire population, but indicative of the DL model’s prediction for a single representative case. The darker areas in each trace on GradCAM denote the areas with the most contribution to DL model’s diagnostic prediction. PR intervals and QRS complexes in STEMI, T waves in NSTEMI, QRS complexes in PHTN, VT beats in patients with non-sustained VT, QRS complexes in AS, p waves in AVB, and ST segment region in HF contributed the most to the diagnosis of each condition. AS aortic stenosis, AVB atrioventricular block, DL deep learning, ECG electrocardiogram, HF heart failure, NSTEMI non-ST-elevation myocardial infarction, STEMI ST-elevation myocardial infarction, PHTN pulmonary hypertension, VT ventricular tachycardia.

**Fig. 5:**
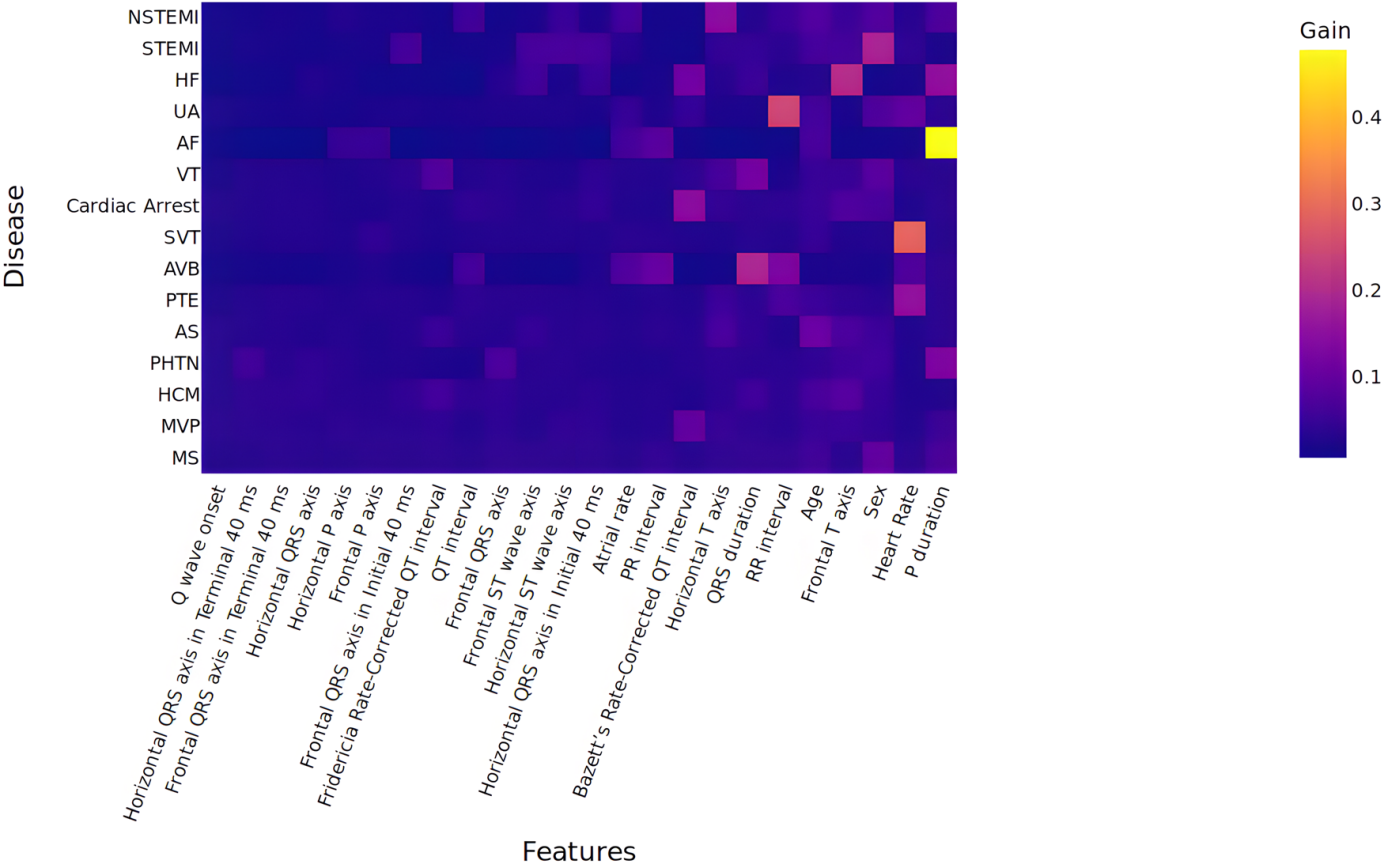
Heatmap of feature importance analyses of XGBoost models with ECG measurements, age, and sex. Information gain-based feature importance for various cardiovascular conditions with XGBoost models based on ECG measurements showed substantial information gain with P-duration for prediction of AF, heart rate for SVT, RR interval for UA etc. ECG electrocardiogram. Abbreviations for ECG measurements and diseases are provided in Supplementary Tables 8 and 9.

Figure 4 depicts the results of GradCAM, highlighting areas of ECG with higher contribution and relevance towards the model’s prediction of different CV conditions (see Supplementary Figure 2 for a full list of all 15 diagnoses). Notably, the regions that contributed the most to the diagnosis were: PR intervals and QRS complexes in STEMI, T waves in NSTEMI, QRS complexes in PHTN, VT beats in patients with non-sustained VT, QRS complexes in AS, p waves in AVB, and ST segment region in HF. Figure 5 shows feature importance analyses of XGB model based on ECG measurements, depicting substantial information gain with P-duration for prediction of AF, heart rate for SVT, RR interval for UA, QRS duration for AVB, frontal T axis for HF, horizontal T axis for NSTEMI, Bazett’s rate-corrected QT interval for CA, etc.

## Discussion

In this large, population-level study with linked administrative health records including millions of ECGs, we developed and validated ML-based prediction models for diagnosing common CV conditions including those previously not explored in ECG-based prediction studies and found both DL and XGB models demonstrated good-to-excellent prediction performance and that DL models performed better than XGB models for most of the studied CV conditions.

Previous studies using AI-enabled ECG diagnosis have shown that ML and DL models can accurately recognize ECG rhythm and morphological abnormalities in ECG, however, they have not provided insights into performance for detecting cardiac conditions that are not routinely diagnosed via ECG^26^. Our study demonstrates how standard ML techniques can learn models that can use the simple and easy-to-obtain 12-lead ECG to accurately predict not only CV conditions but also disorders not conventionally diagnosed using ECGs. These models are potential tools for early screening of CV conditions, particularly those that place considerable burden on the healthcare system, and may help more proximal identification of clinically important CV conditions^27–29^. More importantly, the use of these automated classification systems could enhance access to care in remote areas that have limited access to qualified medical and cardiology specialists. Further investigation is required to determine whether automatic ECG-based screening interfaces can be deployed for early management and prevention of disease progression and to provide cost-effective care.

DL models are complex algorithms with millions of parameters, and likely to overfit when trained on small datasets^30–32^. Even when large medical datasets are available, they are usually unlabeled or unannotated, which poses further challenges for supervised ML approaches^33^. Our large Alberta ECG dataset using the gold-standard 12 leads and its linkage to population-level data^25,34^ represents a naturalistic population laboratory with a wide array of demographic and clinical covariates, and hence, provides the ideal setting for developing ECG-based ML algorithms for the prediction of common CV conditions. Additionally, the current study focused on the very first ECG captured in a healthcare episode, to emulate real-life scenarios of a patient’s initial medical contact at the point of care.

Our DL model showed C-index or AUROC levels >80% for 12 out of 15 conditions, and >90% for 4 conditions (i.e., STEMI, MS, HCM, and AVB). The DL model of ECG tracing provided better prediction than the XGB model of ECG routine measurements for the prediction of all included conditions (except for atrioventricular block where both models had comparable excellent performance), with up to 11.8% improvement in performance for mitral valvulopathy and up to 7.3% improvement in performance in detecting myocardial infarction. Importantly, we further evaluated model robustness with respect to any potential biases towards sex groups and ECGs acquired in the presence of cardiac pacing or left ventricular assist device (LVAD), which can complicate ECG interpretation. We found that our DL model remains robust and appears to work equally well when evaluated on patients of either sex or pacing/LVAD. In fact, our models showed even better performance when ECGs with pacemakers were included in the testing data.

Our study has some limitations that require further discussion. First, all ECGs were generated by machines from the same manufacturer (Phillips Intelligence System), which might limit the generalizability and extrapolation of findings to ECGs from other systems. Second, ECG measurements used in the XGB models were provided through Phillips machines, and were not core laboratory-read or human expert-curated. Third, our labels were derived from ICD codes recorded in the ED and hospitalization record. Owing to the nature of data collection in administrative medical records, the precise timing of condition’s presentation during a healthcare episode cannot be definitively ascertained. Consequently, in rare instances where an acute condition emerges after the collection of the initial ECG, our prediction task can be interpreted as early detection rather than diagnostic prediction of an existing condition. This distinction is noteworthy, as early detection remains valuable in clinical management, offering insights into potential complications in the near future that can be equally beneficial for inpatient care. Fourth, internal testing, even on a substantial scale, may be considered secondary to external validations. This is primarily because biases inherent to a single health system can be perpetuated due to similarities in patient population, equipment, label generation procedures, and other factors. Unfortunately, we were unable to offer external validation for our multi-label models as there is no appropriate external ECG dataset linked to selected 15 ICD-10-based diagnostic labels. However, the performance of leave-one-hospital-out validation of our DL: ECG, age, sex models demonstrates the robustness of our models across hospitals. Fifth, our study is based on a real-world cohort of patients presenting to emergency departments and hospitals with varying prevalence rates of the diagnoses of interest. The variation in the positive rate of the different labels could explain why predictions of some diagnoses were more accurate than others. We did not augment or manipulate the data as our goal is to eventually deploy these models within electronic medical record systems. Moreover, our training dataset of nearly a million ECGs, had a sufficient number of positive cases, to develop effective predictive models. However, some labels in our models may exhibit PPV that might be lower than optimal, necessitating careful consideration regarding their eligibility for clinical deployment. Sixth, we evaluated our models primarily using AUROC or C-index, a common metric in biomedical literature. However, it has limitations and does not consider misclassification costs of false positives or false negatives. For model deployment, custom evaluations aimed at minimizing expected cost, that consider misclassification costs and other resource allocation factors should be prioritized^35^. Furthermore, despite the black-box nature of some of the ML approaches, we used techniques such as GradCAM analysis of DL models (respectively, SHAP analysis of XGB models) to find ECG patterns (or ECG measurements) that contribute to the diagnosis of common CV conditions.

In conclusion, we demonstrate, using comprehensive linked administrative databases at the population level, that ECG-based DL and XGB prediction models demonstrate good-to-excellent prediction performance in diagnosing common CV conditions. The DL models of ECG tracing provided better prediction accuracy among the studied conditions than the XGB models based on routine ECG measurements. Models performed comparably between different sex groups and in patients with and without pacing or LVAD. Future research is needed to determine how these models can be implemented in clinical practice for early diagnosis and risk stratification.

## Methods

### Data sources

This study was performed in Alberta, Canada, where there is a single-payer healthcare system with universal access and 100% capture of all interactions with the healthcare system.

ECG data was linked with the following administrative health databases using a unique patient health number: (1) Discharge Abstract Database (DAD) containing data on inpatient hospitalizations; (2) National Ambulatory Care Reporting System (NACRS) database of all hospital-based outpatient clinic, and emergency department (ED) visits; and (3) Alberta Health Care Insurance Plan Registry (AHCIP), which provides demographic information.

### ECG data

We used standard 12-lead ECG traces (voltage-time series, sampled at 500 Hz for the duration of 10 seconds for each of 12 leads) and ECG measurements (automatically generated by Philips IntelliSpace ECG system’s built-in algorithm). The ECG measurement included atrial rate, heart rate, RR interval, P wave duration, frontal P axis, horizontal P axis, PR interval, QRS duration, frontal QRS axis in the initial 40 ms, frontal QRS axis in the terminal 40 ms, frontal QRS axis, horizontal QRS axis in the initial 40 ms, horizontal QRS axis in terminal 40 ms, horizontal QRS axis, frontal ST wave axis (equivalent to ST deviation), frontal T axis, horizontal ST wave axis, horizontal T axis, Q wave onset, Fridericia rate-corrected QT interval, QT interval, Bazett’s rate-corrected QT interval.

### Analysis cohort

The study cohort has been described previously^25^. In brief, patients who were hospitalized at 14 sites between February 2007 and April 2020 in Alberta, Canada, and includes 2,015,808 ECGs from 3,336,091 ED visits and 1,071,576 hospitalizations of 260,065 patients. Concurrent healthcare encounters (ED visits and/or hospitalizations) that occurred for a patient within a 48-hour period of each other were considered to be transfers and part of the same healthcare episode. An ECG record was linked to a healthcare episode if the acquisition date was within the timeframe between the admission date and discharge date of an episode. After excluding the ECGs that could not be linked to any episode, ECGs of patients <18 years of age, as well as ECGs with poor signal quality (identified via warning flags generated by the ECG machine manufacturer’s built-in quality algorithm), our analysis cohort contained 1,605,268 ECGs from 748,773 episodes in 244,077 patients (Fig. 1).

### Prediction tasks

We developed and evaluated ECG-based models to predict the probability of a patient being diagnosed with any of 15 specific common CV conditions: AF, SVT, VT, CA, AVB, UA, NSTEMI, STEMI, PTE, HCM, AS, MVP, MS, PHTN, and HF. The conditions were identified based on the record of corresponding International Classification of Diseases, 10th revision (ICD-10) codes in the primary or in any one of 24 secondary diagnosis fields of a healthcare episode linked to a particular ECG (Supplementary Table 5). The validity of ICD coding in administrative health databases has been established previously^36,37^. If an ECG was performed during an ED or inpatient episode, it was considered positive for all diagnoses of interest that were recorded in the episode. Some diagnoses, such as AF, SVT, VT, STEMI, and AVB, which are typically identified through ECGs, were included in the study as positive controls to showcase the effectiveness of our models in detecting ECG-diagnosable conditions.

The goal of the prediction model was to output calibrated probabilities for each of selected 15 conditions. These learned models could use ECGs that were acquired at any time point during a healthcare episode. Note that a single patient visit may involve multiple ECGs. When training the model, we used all ECGs (multiple ECGs belonging to the same episode were included) in the training/development set to maximize learning. However, to evaluate our models, we used only the earliest ECG in a given episode in the test/holdout set, with the goal of producing a prediction system that could be employed at the point of care, when the patient’s first ECG is acquired during an ED visit or hospitalization (See section ‘Evaluation’ below for more details).

We used ResNet-based DL for the information-rich voltage-time series and gradient boosting-based XGB for the ECG measurements^25^. To determine whether demographic features (age and sex) add incremental predictive value to the performance of models trained on ECGs only, we developed and reported the models in the following manner: (a) ECG only (DL: ECG trace); (b) ECG + age, sex (DL: ECG trace, age, sex [which is the primary model presented in this study]); and (c) XGB: ECG measurement, age, sex.

### Learning algorithms

We employed a multi-label classification methodology with binary labels—i.e., presence (yes) or absence (no) for each one of the 15 diagnoses—to estimate the probability of a new patient having each of these conditions. Since the input for the models that used ECG measurements was structured tabular data, we trained gradient-boosted tree ensembles (XGB)^38^ models, whereas we used deep convolutional neural networks for the models with ECG voltage-time series traces. For both XGB and DL models, we used 90% of training data to train the model, and used the remaining 10% as a tuning set to track the performance loss and to “early stop” the training process, to reduce the chance of overfitting^39^. For DL, we learned a single ResNet model for a multi-class multi-label task^10^, which mapped each ECG signal into 15 values, corresponds to the probability of presence of each of the 15 diagnoses. On the other hand, for gradient boosting, we learned 15 distinct binary XGB models, each mapping the ECG signal to the probability for one of the individual labels. The methodological details of our XGB and DL model implementations have been described previously^25^.

### Evaluation and visualization

Evaluation design: we used a 60/40 split on the data for training and evaluation. We divided the overall ECG dataset into random splits of 60% for the model development (which used fivefold internal cross-validation for training and fine-tuning the final models) and the remaining 40% as the holdout set for final external validation. We ensured that ECGs from the same patient were not shared between development and evaluation data or between the train/test folds of internal cross-validation. As mentioned earlier, since we expect the deployment scenario of our prediction system to be at the point of care, we evaluated our models using only the patient’s first ECG in a given episode, which was captured during an ED visit or hospitalization. The number of ECGs, episodes, and patients used in overall data and in experimental splits are presented in Fig. 1 and Supplementary Table 5. In addition to the primary evaluation, we extend our testing to include all ECGs from the holdout set, to demonstrate the versatility of DL model in handling ECGs captured at any point during an episode.

Furthermore, we performed ‘Leave-one-hospital-out validation’ using two large tertiary care hospitals to assess the robustness of our model with respect to distributional differences between the hospital sites. To guarantee complete separation between our training and testing sets, we omitted ECGs of patients admitted to both the training and testing hospitals during the study period, as illustrated in Supplementary Figure 1. Finally, to underscore the applicability of DL model in screening scenarios, we present additional evaluations by consolidating 15 disease labels into a composite prediction, thereby enhancing diagnostic yield^20^.

We reported area under the receiver operating characteristic curve (AUROC, equivalent to C-index) and area under the precision-recall curve (AUPRC). Also, we generated F1 Score, Specificity, Recall, Precision (equivalent to PPV) and Accuracy after binarizing the prediction probabilities into diagnosis/non-diagnosis classes using optimal cut-points derived from the training set Youden’s index^40^. We also used the calibration metric Brier Score^41^ (where a smaller score indicates better calibration) to evaluate whether predicted probabilities agree with observed proportions.

Sex and Pacemaker Subgroups: We investigated our models’ performance in specific patient subgroups, based on the patient’s sex. We also investigated any potential bias with ECGs captured in the presence of cardiac pacing (including pacemaker or implantable cardioverter-defibrillators [ICD]) or ventricular assist devices (VAD) since ECG interpretation can be difficult in these situations, by comparing the model performances in ECGs without pacemakers in the holdout set versus the overall holdout set (including ECGs both with or without pacemakers) (Fig. 1). The diagnosis and procedure codes used for identifying the presence of pacemakers are provided in the Supplementary Table 7.

Model comparisons: For each evaluation, we report the performances from the fivefold internal cross-validation as well as the final performances in the holdout set, using the same training and testing splits for the various modeling scenarios. The performances were compared between models by sampling holdout instances with replacement in pairwise manner, to generate a total of 10,000 bootstrap replicates of pairwise differences in AUROC—i.e., each comparing without pacemakers versus the original. The difference in the model performances was said to be statistically significant if the 95% confidence intervals of the mean pairwise differences in AUROCs did not include the zero value for the compared models.

Visualizations: We used feature importance values based on information gained to identify the ECG measurements that were key contributors to the diagnosis prediction in the XGB models. Further, we visualized the gradient activation maps that contributed to the model’s prediction of diagnosis in our DL models using Gradient-weighted Class Activation Mapping (GradCAM)^42^ on the last convolutional layer. Also, we used feature importance values based on information gain to identify the ECG measurements that were key contributors to the diagnosis prediction in the XGB models.

### Supplementary information

Supplemental Material

## Data Availability

The data underlying this article was provided by Alberta Health Services under the terms of a research agreement. Inquiries respecting access to the data can be made directly to them. We have included an ECG dataset that is artificially generated for the purpose of code demonstration only. They are not expected to accurately represent real ECG signals, or the label distributions. The demo dataset is openly available, and can be downloaded at https://figshare.com/s/b593e8d7bfe7cd8500b1.

## Code availability

The code base for training the deep learning models used in this study is available at: https://figshare.com/s/b593e8d7bfe7cd8500b1.

## References

[1] Tison GH, Zhang J, Delling FN, Deo RC (2019). Automated and interpretable patient ECG profiles for disease detection, tracking, and discovery. Circ. Cardiovasc. Qual. Outcomes.

[2] Attia ZI (2019). Age and sex estimation using artificial intelligence from standard 12-lead ECGs. Circ. Arrhythm. Electrophysiol..

[3] Attia ZI (2019). Screening for cardiac contractile dysfunction using an artificial intelligence-enabled electrocardiogram. Nat. Med..

[4] Attia ZI (2019). An artificial intelligence-enabled ECG algorithm for the identification of patients with atrial fibrillation during sinus rhythm: a retrospective analysis of outcome prediction. Lancet.

[5] Kwon J-M (2020). Comparing the performance of artificial intelligence and conventional diagnosis criteria for detecting left ventricular hypertrophy using electrocardiography. Europace.

[6] Sraitih, M., Jabrane, Y. & Hajjam El Hassani, A. An automated system for ECG arrhythmia detection using machine learning techniques. *J. Clin. Med. Res*. 1**0**, 5450 (2021). 10.3390/jcm10225450 PMC8618527 34830732

[7] Gustafsson S (2022). Development and validation of deep learning ECG-based prediction of myocardial infarction in emergency department patients. Sci. Rep..

[8] Wu L (2022). Deep learning networks accurately detect st-segment elevation myocardial infarction and culprit vessel. Front Cardiovasc. Med..

[9] Al-Zaiti SS (2023). Machine learning for ECG diagnosis and risk stratification of occlusion myocardial infarction. Nat. Med..

[10] Ribeiro AH (2020). Automatic diagnosis of the 12-lead ECG using a deep neural network. Nat. Commun..

[11] Isasi I (2019). A robust machine learning architecture for a reliable ECG rhythm analysis during CPR. Conf. Proc. IEEE Eng. Med. Biol. Soc..

[12] Elola, A. et al. Deep neural networks for ECG-based pulse detection during out-of-hospital cardiac arrest. *Entropy* **21**, 305 (2019). 10.3390/e21030305 PMC7514786 33267020

[13] Choi J (2022). Deep learning of ECG waveforms for diagnosis of heart failure with a reduced left ventricular ejection fraction. Sci. Rep..

[14] Raghu A (2023). ECG-guided non-invasive estimation of pulmonary congestion in patients with heart failure. Sci. Rep..

[15] Somani SS (2022). Development of a machine learning model using electrocardiogram signals to improve acute pulmonary embolism screening. Eur. Heart J. Digit Health.

[16] Valente Silva B, Marques J, Nobre Menezes M, Oliveira AL, Pinto FJ (2023). Artificial intelligence-based diagnosis of acute pulmonary embolism: Development of a machine learning model using 12-lead electrocardiogram. Rev. Port. Cardiol..

[17] Hata E (2020). Classification of aortic stenosis using ECG by deep learning and its analysis using grad-CAM. Conf. Proc. IEEE Eng. Med. Biol. Soc..

[18] Goto S (2022). Multinational federated learning approach to train ECG and echocardiogram models for hypertrophic cardiomyopathy detection. Circulation.

[19] Cohen-Shelly M (2021). Electrocardiogram screening for aortic valve stenosis using artificial intelligence. Eur. Heart J..

[20] Ulloa-Cerna AE (2022). rECHOmmend: an ECG-based machine learning approach for identifying patients at increased risk of undiagnosed structural heart disease detectable by echocardiography. Circulation.

[21] Aras MA (2023). Electrocardiogram detection of pulmonary hypertension using deep learning. J. Card. Fail..

[22] Liu C-M (2022). Artificial intelligence-enabled electrocardiogram improves the diagnosis and prediction of mortality in patients with pulmonary hypertension. JACC Asia.

[23] Chen L, Fu G, Jiang C (2023). Deep learning-derived 12-lead electrocardiogram-based genotype prediction for hypertrophic cardiomyopathy: a pilot study. Ann. Med..

[24] Ko W-Y (2020). Detection of hypertrophic cardiomyopathy using a convolutional neural network-enabled electrocardiogram. J. Am. Coll. Cardiol..

[25] Sun W (2023). Towards artificial intelligence-based learning health system for population-level mortality prediction using electrocardiograms. NPJ Digit. Med..

[26] Liu X, Wang H, Li Z, Qin L (2021). Deep learning in ECG diagnosis: a review. Knowl.-Based Syst..

[27] Mant J (2007). Accuracy of diagnosing atrial fibrillation on electrocardiogram by primary care practitioners and interpretative diagnostic software: analysis of data from screening for atrial fibrillation in the elderly (SAFE) trial. BMJ.

[28] Veronese G (2016). Emergency physician accuracy in interpreting electrocardiograms with potential ST-segment elevation myocardial infarction: is it enough?. Acute Card. Care.

[29] Tran DT (2016). The current and future financial burden of hospital admissions for heart failure in Canada: a cost analysis. CMAJ Open.

[30] Somani S (2021). Deep learning and the electrocardiogram: review of the current state-of-the-art. Europace.

[31] Clifford, G. D. et al. AF classification from a short single lead ECG recording: the PhysioNet/computing in cardiology challenge 2017. *Comput. Cardiol*. **4****4**, 10.22489/CinC.2017.065-469 (2017). 10.22489/CinC.2017.065-469 PMC5978770 29862307

[32] Hannun AY (2019). Cardiologist-level arrhythmia detection and classification in ambulatory electrocardiograms using a deep neural network. Nat. Med..

[33] Sun, W. et al. Improving ECG-based COVID-19 diagnosis and mortality predictions using pre-pandemic medical records at population-scale. *In:* Time series for health at NeurIPS. 10.48550/arXiv.2211.10431. (2022).

[34] Sun, W. et al. ECG for high-throughput screening of multiple diseases: Proof-of-concept using multi-diagnosis deep learning from population-based datasets. *In:* Medical imaging meets NeurIPS. 10.48550/arXiv.2210.06291. (2022).

[35] Drummond C, Holte RC (2006). Cost curves: an improved method for visualizing classifier performance. Mach. Learn..

[36] Quan H (2008). Assessing validity of ICD-9-CM and ICD-10 administrative data in recording clinical conditions in a unique dually coded database. Health Serv. Res..

[37] Quan H (2005). Coding algorithms for defining comorbidities in ICD-9-CM and ICD-10 administrative data. Med. Care.

[38] Chen, T. & Guestrin, C. XGBoost: a scalable tree boosting system. *In:* Proceedings of the 22nd ACM SIGKDD international conference on knowledge discovery and data mining, 785–794 (Association for Computing Machinery, 2016).

[39] Prechelt, L. Early stopping — but when? *In:* neural networks: tricks of the trade: Second Edition (eds. Montavon, G., Orr, G. B. & Müller, K.-R.) 53–67 (Springer Berlin Heidelberg, 2012).

[40] Youden WJ (1950). Index for rating diagnostic tests. Cancer.

[41] Brier GW (1950). Verification of forecasts expressed in terms of probability. Mon. Weather Rev..

[42] Selvaraju, R. R. et al. Grad-CAM: visual explanations from deep networks via gradient-based localization. *In:* 2017 IEEE International Conference on Computer Vision (ICCV) 618–626 (2017).

[43] Moons KGM (2015). Transparent Reporting of a multivariable prediction model for Individual Prognosis or Diagnosis (TRIPOD): explanation and elaboration. Ann. Intern. Med..

