# Supplementary material for: Development and validation of machine learning algorithms based on electrocardiograms for cardiovascular diagnoses at the population level
Source: NPJ Digit Med. 2024 May 18;7:133. doi: 10.1038/s41746-024-01130-8 (PMC11102430; doi:10.1038/s41746-024-01130-8)
Supplement: Supplementary file 1 — Supplemental Material [file 41746_2024_1130_MOESM1_ESM.pdf]

Supplementary Figures

Supplementary Figure 1. Flowchart of the study design showing the sample sizes for different splits and outcomes for leave-one-hospital out validation for the Hospitals H1 (top panel) and H2 (bottom panel).

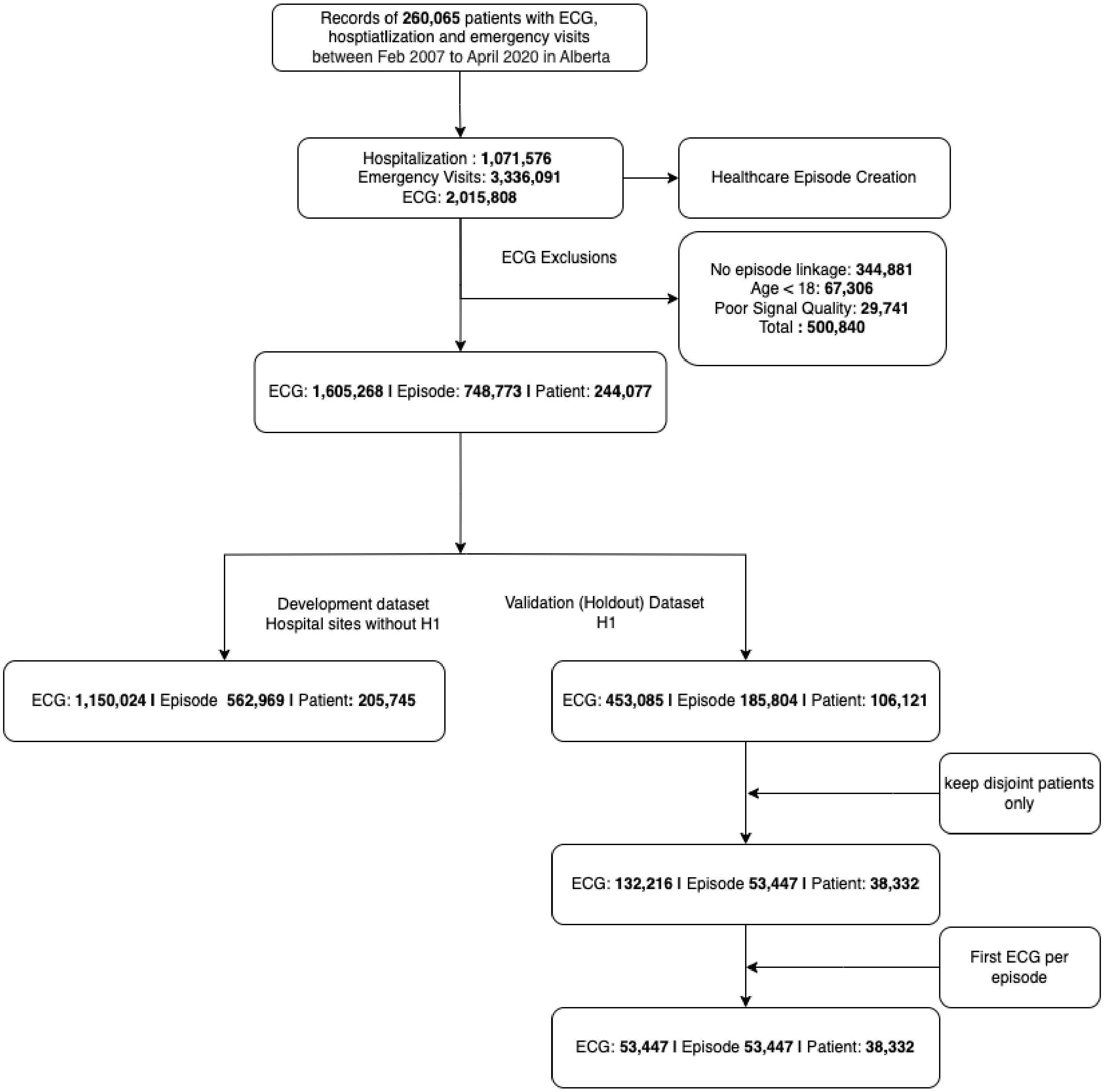

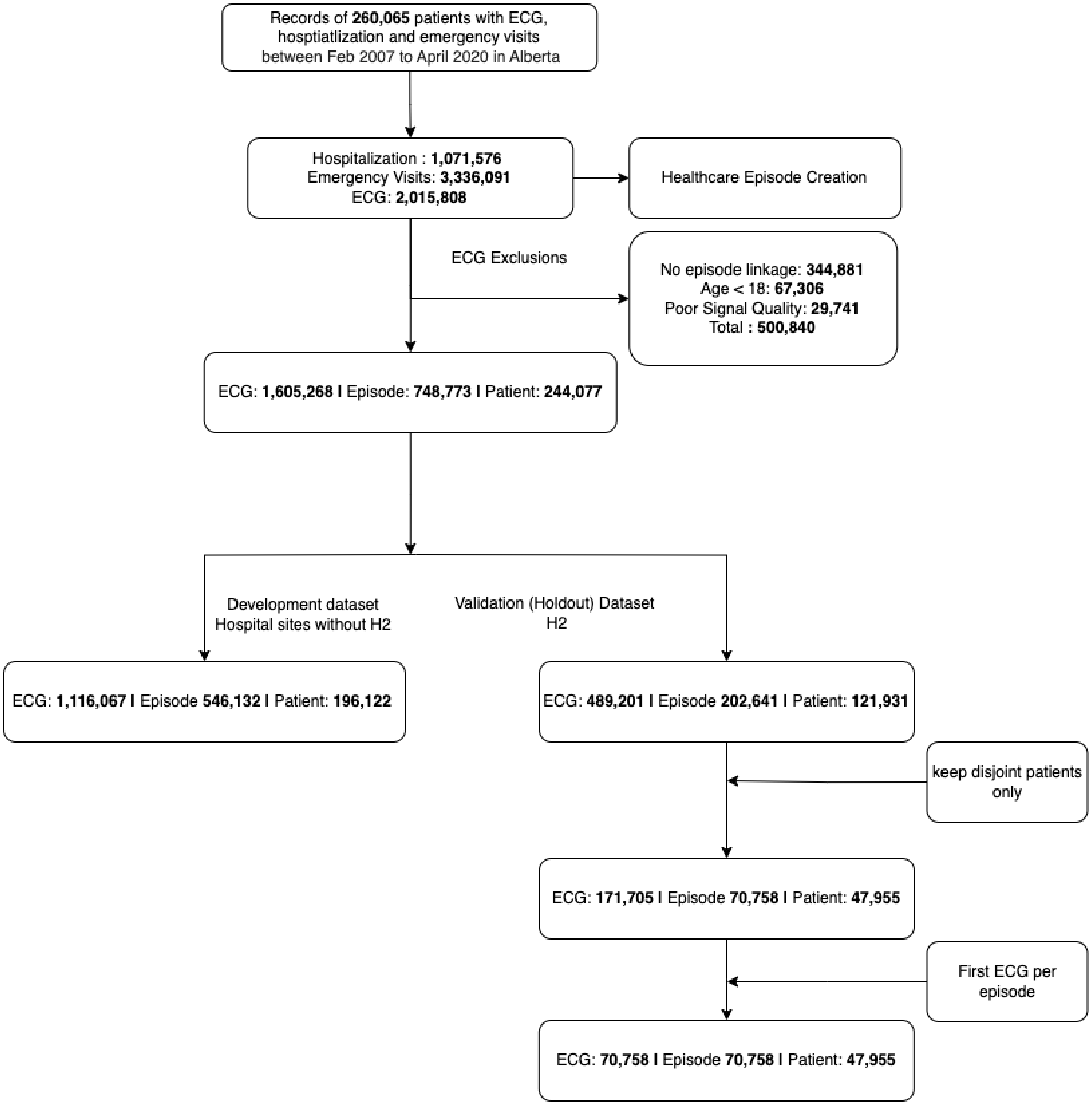
Records of **260,065** patients with ECG, hosptiatlization and emergency visits between Feb 2007 to April 2020 in Alberta
Hospitalization : **1,071,576**
Emergency Visits: **3,336,091**
ECG: **2,015,808**
Healthcare Episode Creation
ECG Exclusions
No episode linkage: **344,881**
Age < 18: **67,306**
Poor Signal Quality: **29,741**
Total **: 500,840**
ECG: **1,605,268 I** Episode: **748,773 I** Patient: **244,077**
Development dataset
Hospital sites without H2
Validation (Holdout) Dataset
H2
ECG: **1,116,067 I** Episode **546,132 I** Patient: **196,122**
ECG: **489,201 I** Episode **202,641 I** Patient: **121,931**
keep disjoint patients only
ECG: **171,705 I** Episode **70,758 I** Patient: **47,955**
First ECG per episode
ECG: **70,758 I** Episode **70,758 I** Patient: **47,955**

Supplementary Figure 2. The GradCAM plots for the DL: ECG, age, sex model in diagnosis of 15 different cardiovascular conditions.

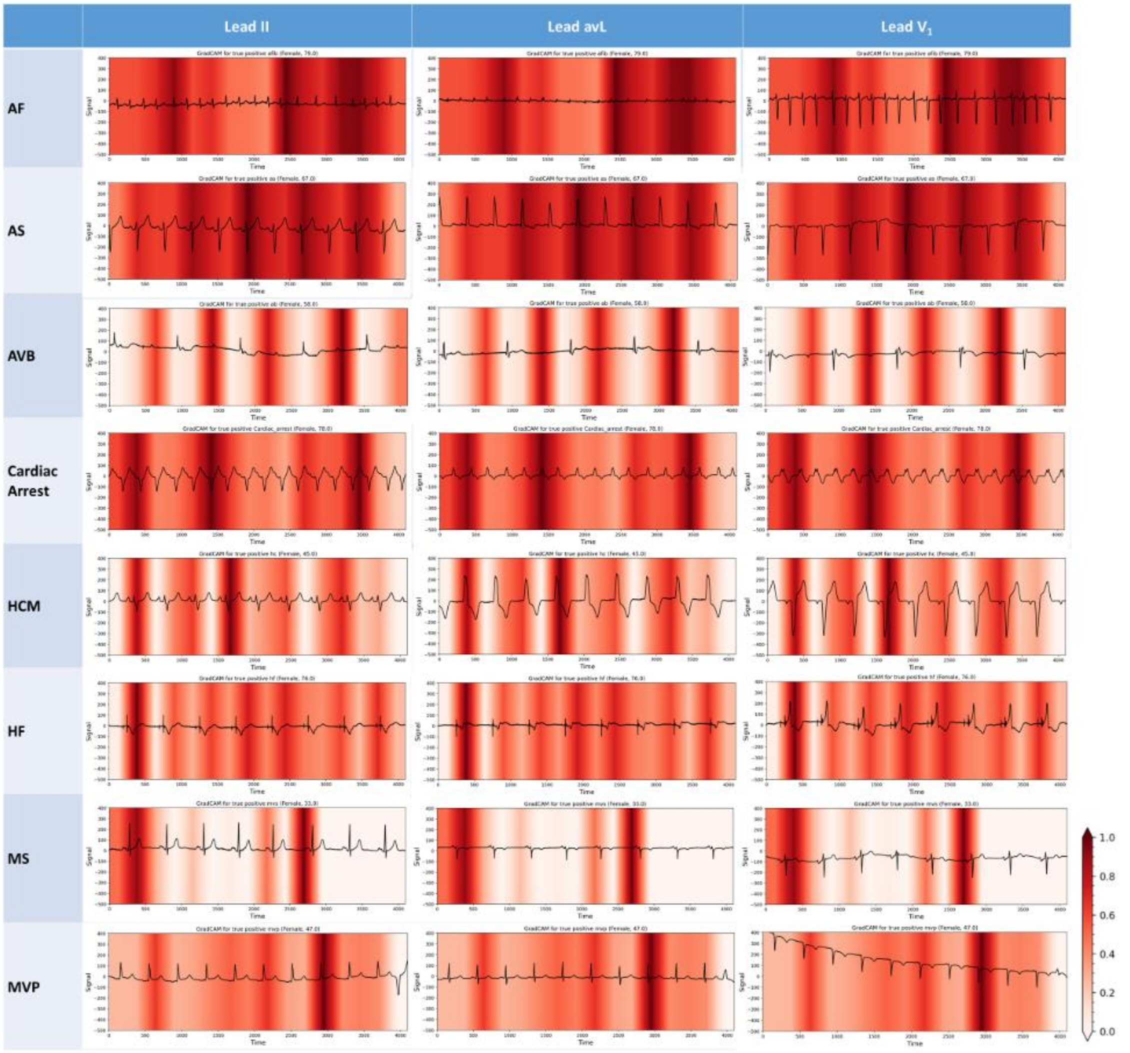

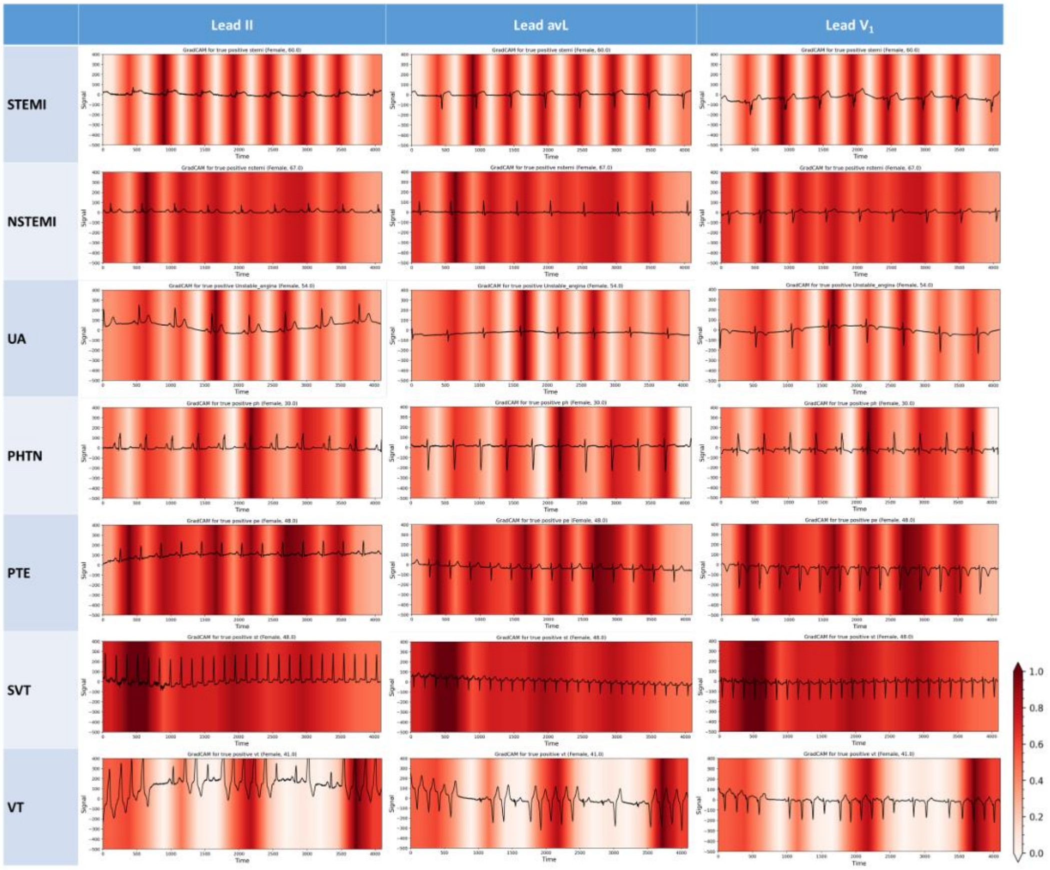

Representative ECG traces were chosen with the 15 diagnoses labels. The darker areas in each trace on GradCAM denote the areas with the most contribution to DL model's diagnostic prediction. AF: atrial fibrillation; AS: Aortic stenosis; AVB: Atrioventricular block; DL: deep learning (ResNet); ECG: electrocardiogram; HCM: hypertrophic cardiomyopathy; HF: heart failure; MS: mitral stenosis; MVP: mitral valve prolapse; NSTEMI: Non-ST-elevation myocardial infarction; STEMI: ST-elevation myocardial infarction; SVT: supraventricular tachycardia; PHTN: pulmonary hypertension; PTE: Pulmonary thromboembolism; UA: unstable angina; VT: ventricular tachycardia.

Supplementary Tables

Supplementary Table 1: Characteristics of patient cohorts used in the study.

	Full Data (n= 1,605,268)	Development set (n = 964,741)	Holdout set (n = 640,527)	First ECG per episode in holdout set (n = 297,773)
Age (years)	65.80 ± 17.25	65.77 ± 17.22	65.85 ± 17.29	64.66 ± 18.52
Sex (Male in %)	56.73	56.81	56.6	52.72
ECG measurements				
Atrial rate	85.60 ± 46.15	85.56 ± 46.11	85.67 ± 46.20	84.89 ± 42.06
P duration	155.92 ± 116.60	156.00 ± 116.39	155.79 ± 116.91	158.93 ± 113.25
RR interval	790.81 ± 213.11	790.90 ± 212.63	790.68 ± 213.83	782.79 ± 201.37
Q wave onset	508.84 ± 6.51	508.82 ± 6.29	508.87 ± 6.82	508.99 ± 6.21
Fridericia rate-corrected QT interval	434.86 ± 38.05	434.96 ± 38.04	434.71 ± 38.07	431.33 ± 35.20
Heart Rate	81.64 ± 23.22	81.61 ± 23.18	81.69 ± 23.28	81.91 ± 22.04
PR interval	169.34 ± 38.46	169.44 ± 37.66	169.18 ± 39.65	167.89 ± 39.12
QRS duration	101.36 ± 24.26	101.40 ± 24.23	101.31 ± 24.30	99.66 ± 23.04
QT interval	399.81 ± 54.83	399.94 ± 54.74	399.63 ± 54.96	395.40 ± 51.06
Bazett's Rate-Corrected QT interval	455.02 ± 40.09	455.10 ± 40.09	454.89 ± 40.09	451.86 ± 37.18
Frontal P axis	44.85 ± 35.52	44.81 ± 35.41	44.91 ± 35.69	45.43 ± 34.22
Frontal QRS axis in Initial 40 ms	27.50 ± 46.30	27.44 ± 46.37	27.59 ± 46.20	28.69 ± 43.58
Frontal QRS axis in Terminal 40 ms	45.36 ± 88.15	45.74 ± 88.28	44.80 ± 87.96	45.41 ± 87.24
Frontal QRS axis	19.98 ± 54.37	20.04 ± 54.38	19.88 ± 54.37	21.07 ± 52.31
Frontal ST wave axis	90.94 ± 88.23	90.98 ± 88.07	90.87 ± 88.48	84.27 ± 85.68
Frontal T axis	55.70 ± 67.76	55.48 ± 67.60	56.03 ± 68.00	50.44 ± 59.22
Horizontal P axis	20.69 ± 47.30	20.63 ± 47.14	20.77 ± 47.52	19.43 ± 44.76
Horizontal QRS axis in Initial 40 ms	27.79 ± 48.39	27.86 ± 48.17	27.69 ± 48.71	29.18 ± 43.86
Horizontal QRS axis in Terminal 40 ms	34.10 ± 129.50	33.94 ± 129.40	34.35 ± 129.66	33.33 ± 128.73
Horizontal QRS axis	-0.91 ± 78.19	-1.11 ± 77.91	-0.61 ± 78.62	-1.44 ± 74.31
Horizontal ST wave axis	97.02 ± 64.99	96.98 ± 65.00	97.09 ± 64.97	93.18 ± 60.92
Horizontal T axis	64.46 ± 58.98	64.27 ± 58.96	64.73 ± 59.01	57.77 ± 51.43

Comorbidities				
**Peripheral vascular disease**	33,518 (2.09%)	19,714 (2.04%)	13,804 (2.16%)	4305 (1.45%)
**Cerebrovascular disease**	54,349 (3.39%)	33,191 (3.44%)	21,158 (3.30%)	9840 (3.30%)
**Myocardial infarction**	350,859 (21.86%)	210,275 (21.80%)	140,584 (21.95%)	26,649 (8.95%)
**Hypertension**	133,963 (8.35%)	80,037 (8.30%)	53,926 (8.42%)	21,036 (7.06%)
**Dementia**	31,764 (1.98%)	19,215 (1.99%)	12,549 (1.96%)	5641 (1.89%)
**Chronic pulmonary disease**	120,260 (7.49%)	71,860 (7.45%)	48,400 (7.56%)	23,955 (8.04%)
**Diabetes mellitus**	163,262 (10.17%)	96,924 (10.05%)	66,338 (10.36%)	25,231 (8.47%)
**Renal disease**	20,268 (1.26%)	12,062 (1.25%)	8206 (1.28%)	3354 (1.13%)
**Liver disease**	18,905 (1.18%)	11,707 (1.21%)	7198 (1.12%)	3663 (1.23%)
**Cancer**	93,946 (5.85%)	55,632 (5.77%)	38,314 (5.98%)	17,758 (5.96%)

ECG measurements and comorbidities are expressed in terms of the number of ECGs.
ECG: electrocardiogram; N: number.

Supplementary Table 2: Evaluation of DL: ECG and XGB: ECG, age, sex model performances expressed in mean (95% confidence interval) percentage.

	Model	AUROC	AUPRC	F1 Score	Specificity	Recall	Precision	Accuracy	Brier Score
**Non-ST-elevation myocardial infarction (NSTEMI)**	DL:ECG	81.734 (81.730-81.739)	38.564 (38.558-38.569)	22.026 (22.021-22.030)	84.918 (84.916-84.919)	62.402 (62.393-62.411)	13.373 (13.370-13.376)	84.108 (84.106-84.109)	3.741
**ST-elevation myocardial infarction (STEMI)**	DL:ECG	95.182 (95.178-95.185)	54.512 (54.505-54.519)	40.328 (40.320-40.337)	95.754 (95.753-95.755)	81.942 (81.931-81.952)	26.747 (26.740-26.753)	95.498 (95.497-95.498)	1.116
**Heart failure**	DL:ECG	88.443 (88.441-88.445)	55.739 (55.735-55.742)	46.612 (46.608-46.616)	84.714 (84.712-84.715)	75.465 (75.460-75.470)	33.720 (33.717-33.724)	83.850 (83.848-83.851)	6.443
**Unstable angina**	DL:ECG	78.823 (78.816-78.830)	34.907 (34.899-34.915)	5.988 (5.985-5.990)	76.243 (76.242-76.245)	66.292 (66.276-66.308)	3.135 (3.134-3.137)	76.129 (76.128-76.131)	1.31
**Atrial fibrillation**	DL:ECG	87.938 (87.936-87.940)	58.819 (58.816-58.822)	51.145 (51.141-51.149)	84.101 (84.100-84.103)	76.547 (76.543-76.552)	38.402 (38.398-38.405)	83.235 (83.234-83.237)	7.183
**Ventricular tachycardia**	DL:ECG	82.712 (82.701-82.722)	29.905 (29.893-29.917)	6.191 (6.187-6.194)	89.932 (89.931-89.933)	56.271 (56.248-56.293)	3.276 (3.274-3.278)	89.729 (89.728-89.731)	0.653
**Cardiac arrest**	DL:ECG	80.630 (80.622-80.639)	29.456 (29.446-29.466)	7.894 (7.890-7.898)	88.238 (88.237-88.239)	54.218 (54.200-54.236)	4.257 (4.255-4.259)	87.913 (87.912-87.914)	0.953
**Supraventricular tachycardia**	DL:ECG	75.114 (75.103-75.126)	29.265 (29.254-29.275)	4.339 (4.337-4.341)	81.782 (81.781-81.783)	55.943 (55.923-55.964)	2.257 (2.256-2.258)	81.589 (81.588-81.591)	0.701

**Atrioventricular block**	DL:ECG	89.172 (89.165-89.178)	39.093 (39.084-39.101)	11.504 (11.499-11.508)	89.281 (89.279-89.282)	71.651 (71.635-71.667)	6.254 (6.251-6.257)	89.106 (89.105-89.107)	0.872
**Pulmonary embolism**	DL:ECG	68.798 (68.791-68.806)	31.844 (31.837-31.851)	5.392 (5.390-5.394)	66.321 (66.319-66.322)	60.230 (60.216-60.244)	2.822 (2.821-2.823)	66.223 (66.222-66.225)	1.573
**Aortic stenosis**	DL:ECG	85.123 (85.116-85.129)	36.350 (36.341-36.358)	8.020 (8.017-8.023)	83.330 (83.329-83.331)	68.095 (68.078-68.111)	4.261 (4.259-4.263)	83.166 (83.164-83.167)	1.051
**Pulmonary hypertension**	DL:ECG	83.963 (83.957-83.969)	36.519 (36.512-36.527)	9.635 (9.631-9.638)	83.158 (83.157-83.160)	67.411 (67.397-67.426)	5.188 (5.186-5.190)	82.946 (82.944-82.947)	1.343
**Hypertrophic cardiomyopathy**	DL:ECG	84.253 (84.230-84.276)	29.611 (29.587-29.636)	2.401 (2.398-2.404)	93.577 (93.576-93.578)	57.939 (57.890-57.987)	1.226 (1.224-1.227)	93.528 (93.527-93.529)	0.141
**Mitral valve prolapse**	DL:ECG	83.897 (83.889-83.906)	33.451 (33.440-33.462)	5.324 (5.321-5.327)	85.265 (85.263-85.266)	63.889 (63.868-63.910)	2.778 (2.776-2.779)	85.125 (85.124-85.126)	0.681
**Mitral valve stenosis**	DL:ECG	86.690 (86.669-86.712)	32.438 (32.410-32.466)	1.106 (1.105-1.108)	89.329 (89.328-89.330)	64.285 (64.229-64.341)	0.558 (0.557-0.559)	89.306 (89.305-89.307)	0.102
**Non-ST-elevation myocardial infarction (NSTEMI)**	XGB:ECG,AGE,SEX	75.098 (75.093-75.102)	35.729 (35.724-35.734)	14.482 (14.479-14.485)	74.151 (74.150-74.153)	61.887 (61.878-61.896)	8.201 (8.199-8.203)	73.710 (73.708-73.712)	3.734
**ST-elevation myocardial infarction (STEMI)**	XGB:ECG,AGE,SEX	88.380 (88.375-88.385)	41.489 (41.482-41.495)	17.303 (17.298-17.307)	87.379 (87.378-87.381)	72.649 (72.637-72.661)	9.821 (9.818-9.824)	87.106 (87.105-87.107)	1.658
**Heart failure**	XGB:ECG,AGE,SEX	84.471 (84.469-84.473)	51.633 (51.630-51.637)	39.488 (39.484-39.491)	79.357 (79.355-79.358)	73.882 (73.876-73.887)	26.945 (26.941-26.948)	78.845 (78.844-78.847)	7.057

**Unstable angina**	XGB:ECG,AGE,SEX	77.042 (77.035-77.049)	34.318 (34.310-34.326)	5.487 (5.485-5.489)	74.278 (74.276-74.279)	65.376 (65.360-65.392)	2.864 (2.863-2.865)	74.176 (74.174-74.177)	1.164
**Atrial fibrillation**	XGB:ECG,AGE,SEX	82.582 (82.580-82.585)	50.013 (50.009-50.017)	44.391 (44.387-44.395)	85.537 (85.536-85.539)	60.391 (60.386-60.396)	35.094 (35.090-35.098)	82.655 (82.653-82.656)	8.468
**Ventricular tachycardia**	XGB:ECG,AGE,SEX	82.441 (82.430-82.452)	32.412 (32.400-32.424)	5.263 (5.260-5.266)	86.740 (86.738-86.741)	61.846 (61.824-61.869)	2.748 (2.747-2.750)	86.590 (86.588-86.591)	0.599
**Cardiac arrest**	XGB:ECG,AGE,SEX	76.871 (76.862-76.880)	28.731 (28.721-28.740)	5.717 (5.715-5.720)	83.264 (83.262-83.265)	54.004 (53.985-54.022)	3.018 (3.017-3.020)	82.984 (82.983-82.985)	0.964
**Supraventricular tachycardia**	XGB:ECG,AGE,SEX	72.064 (72.052-72.077)	25.718 (25.707-25.729)	4.417 (4.415-4.420)	84.524 (84.523-84.525)	48.740 (48.719-48.761)	2.314 (2.312-2.315)	84.257 (84.255-84.258)	0.687
**Atrioventricular block**	XGB:ECG,AGE,SEX	90.111 (90.105-90.117)	40.301 (40.292-40.309)	10.655 (10.651-10.659)	87.765 (87.764-87.767)	74.613 (74.598-74.629)	5.737 (5.735-5.740)	87.635 (87.634-87.637)	0.884
**Pulmonary embolism**	XGB:ECG,AGE,SEX	66.302 (66.295-66.310)	27.976 (27.968-27.983)	5.244 (5.242-5.246)	70.001 (69.999-70.002)	52.431 (52.417-52.445)	2.760 (2.759-2.761)	69.720 (69.718-69.721)	1.566
**Aortic stenosis**	XGB:ECG,AGE,SEX	79.625 (79.618-79.632)	32.096 (32.088-32.105)	6.085 (6.082-6.088)	80.062 (80.060-80.063)	60.564 (60.548-60.581)	3.203 (3.202-3.205)	79.852 (79.850-79.853)	1.061
**Pulmonary hypertension**	XGB:ECG,AGE,SEX	79.312 (79.305-79.318)	32.420 (32.412-32.428)	7.626 (7.623-7.629)	80.596 (80.595-80.597)	60.232 (60.217-60.247)	4.071 (4.069-4.072)	80.321 (80.320-80.323)	1.29
**Hypertrophic cardiomyopathy**	XGB:ECG,AGE,SEX	85.550 (85.528-85.572)	31.093 (31.069-31.118)	2.434 (2.431-2.437)	93.337 (93.337-93.338)	60.891 (60.844-60.939)	1.242 (1.240-1.243)	93.293 (93.292-93.294)	0.13
**Mitral valve prolapse**	XGB:ECG,AGE,SEX	75.921 (75.910-75.931)	29.391 (29.380-29.402)	3.585 (3.583-3.587)	80.207 (80.206-80.209)	56.647 (56.625-56.669)	1.851 (1.850-1.852)	80.053 (80.051-80.054)	0.655

**Mitral valve stenosis**	XGB:ECG,AGE,SEX	78.456 (78.428-78.485)	19.202 (19.173-19.230)	0.994 (0.992-0.996)	93.036 (93.035-93.037)	37.842 (37.786-37.899)	0.504 (0.503-0.505)	92.984 (92.983-92.985)	0.092

AUPRC: Area under the precision-recall curve; AUROC: Area under the receiver operating curve; DL: Deep learning; ECG: electrocardiogram; XGB: XGBoost.

Supplementary Table 3: Independent hospital evaluation of DL: ECG, Age, Sex model performances, expressed in mean percentage.

**H1**	**AUROC**	**AURPC**	**F1 Score**	**Precision**	**Recall**	**Specificity**	**Accuracy**	**Brier Score**	**Positive (N)**	**Negative (N)**
**Non-ST elevation (NSTEMI) myocardial infarction**	85.74%	48.21%	34.62%	22.81%	71.78%	83.22%	82.48%	5.06%	3448	49999
**ST elevation (STEMI) myocardial infarction**	95.62%	63.97%	55.49%	40.89%	86.31%	92.80%	92.44%	2.49%	2914	50533
**Heart failure**	90.78%	55.68%	48.36%	36.04%	73.47%	90.24%	89.07%	4.57%	3717	49730
**Unstable angina**	81.92%	39.49%	9.42%	5.03%	73.48%	74.76%	74.74%	1.87%	954	52493
**Atrial fibrillation**	90.20%	59.03%	54.16%	43.20%	72.57%	91.29%	89.72%	4.87%	4466	48981
**Ventricular Tachycardia**	82.95%	33.02%	7.83%	4.18%	61.56%	89.05%	88.84%	0.78%	411	53036
**Cardiac arrest**	84.19%	36.20%	16.12%	9.26%	62.39%	87.60%	87.10%	1.73%	1061	52386
**Supraventricular tachycardia**	76.29%	30.22%	3.61%	1.87%	58.29%	79.06%	78.92%	0.63%	362	53085
**Atrioventricular block**	91.44%	43.34%	20.28%	11.74%	74.61%	92.53%	92.30%	0.90%	701	52746
**Pulmonary embolism**	74.13%	34.24%	7.31%	3.88%	63.95%	70.27%	70.16%	1.73%	982	52465
**Aortic Stenosis**	85.84%	31.14%	6.22%	3.28%	58.75%	89.57%	89.38%	0.62%	320	53127
**Pulmonary Hypertension**	86.97%	38.10%	12.21%	6.70%	69.10%	87.22%	86.99%	1.24%	699	52748
**Hypertrophic Cardiomyopathy**	91.78%	35.97%	3.43%	1.76%	70.15%	95.08%	95.04%	0.13%	67	53380
**Mitral Valve Prolapse**	84.01%	30.83%	8.33%	4.49%	56.78%	89.07%	88.78%	0.88%	479	52968
**Mitral Valve Stenosis**	90.99%	41.39%	2.24%	1.14%	81.63%	93.47%	93.46%	0.09%	49	53398

H2	AUROC	AURPC	F1 Score	Precision	Recall	Specificity	Accuracy	Brier Score	Positive (N)	Negative (N)
Non-ST elevation (NSTEMI)_myocardial infarction	82.81%	41.80%	23.31%	14.06%	68.14%	80.77%	80.22%	4.25%	3120	67638
ST elevation (STEMI) myocardial infarction	95.12%	58.62%	46.10%	31.62%	85.09%	93.18%	92.89%	1.86%	2528	68230
Heart failure	88.60%	56.23%	48.41%	35.99%	73.93%	85.80%	84.65%	6.38%	6890	63868
Unstable angina	79.91%	38.09%	7.11%	3.74%	72.06%	74.00%	73.97%	1.42%	977	69781
Atrial fibrillation	86.51%	59.09%	54.86%	46.15%	67.62%	87.58%	84.87%	8.03%	9618	61140
Ventricular Tachycardia	83.15%	38.41%	7.89%	4.17%	72.30%	79.33%	79.24%	1.20%	870	69888
Cardiac Arrest	79.50%	36.96%	9.89%	5.34%	67.99%	77.20%	77.03%	1.92%	1312	69446
Supraventricular Tachycardia	70.50%	27.67%	4.55%	2.38%	52.44%	76.03%	75.77%	1.05%	778	69980
Atrioventricular Block	90.12%	42.17%	16.56%	9.31%	74.63%	88.11%	87.89%	1.30%	1139	69619
Pulmonary Embolism	72.53%	33.14%	7.35%	3.91%	61.65%	70.55%	70.38%	1.79%	1348	69410
Aortic Stenosis	81.81%	33.62%	17.74%	10.56%	55.39%	85.92%	85.03%	2.74%	2060	68698
Pulmonary Hypertension	85.23%	39.32%	14.85%	8.31%	69.68%	83.45%	83.16%	1.89%	1491	69267
Hypertrophic Cardiomyopathy	87.86%	35.98%	3.83%	1.97%	69.88%	87.20%	87.13%	0.37%	259	70499
Mitral Valve Prolapse	79.98%	31.18%	8.73%	4.73%	56.98%	82.41%	82.03%	1.48%	1067	69691
Mitral Valve Stenosis	88.41%	32.89%	2.22%	1.13%	64.58%	88.45%	88.40%	0.21%	144	70614

AUPRC: area under the precision-recall curve; AUROC: area under the receiver operating curve; DL: deep learning; ECG: electrocardiogram; H1: hospital number 1; H2: hospital number 2

Supplementary Table 4: Evaluation of deep learning: ECG, age, sex model performances for different cardiovascular conditions expressed in mean percentage using all ECGs from the holdout set.

All ECGs from Holdout set	AUROC	AUPRC	F1 Score	Specificity	Recall	Precision	Accuracy	Brier Score
**Non-ST-elevation myocardial infarction**	81.62%	50.00%	38.42%	77.60%	70.59%	26.40%	76.88%	7.85%
**ST-elevation myocardial infarction**	94.35%	62.59%	54.91%	92.06%	83.15%	40.99%	91.51%	3.14%
**Heart failure**	87.45%	62.14%	54.91%	80.12%	78.88%	42.12%	79.92%	9.26%
**Unstable angina**	78.87%	38.22%	12.57%	74.44%	68.68%	6.92%	74.29%	2.55%
**Atrial fibrillation**	86.42%	64.55%	59.57%	82.04%	74.83%	49.49%	80.66%	10.27%
**Ventricular tachycardia**	82.05%	36.64%	12.07%	81.86%	65.99%	6.64%	81.56%	1.84%
**Cardiac arrest**	79.86%	36.45%	13.87%	79.97%	64.21%	7.77%	79.57%	2.45%
**Supraventricular tachycardia**	72.47%	30.07%	6.71%	76.38%	55.89%	3.57%	76.06%	1.46%
**Atrioventricular block**	87.80%	42.52%	21.62%	87.36%	71.60%	12.73%	86.97%	1.86%
**Pulmonary embolism**	71.43%	32.28%	7.64%	70.88%	59.66%	4.08%	70.65%	1.93%
**Aortic stenosis**	83.50%	37.93%	11.40%	80.36%	69.07%	6.21%	80.15%	1.74%
**Pulmonary hypertension**	83.06%	40.28%	12.40%	76.74%	73.18%	6.77%	76.66%	2.13%
**Hypertrophic cardiomyopathy**	84.12%	28.31%	3.61%	92.88%	54.65%	1.86%	92.79%	0.25%
**Mitral valve prolapse**	82.01%	36.00%	9.56%	80.22%	66.32%	5.15%	80.00%	1.59%
**Mitral valve stenosis**	86.50%	30.47%	2.22%	90.37%	59.73%	1.13%	90.31%	0.19%

AUPRC: area under the precision-recall curve; AUROC: area under the receiver operating curve; DL: deep learning; ECG: electrocardiogram

Supplementary Table 5: ICD 10 codes used for the identifying diagnostic labels.

**Diagnoses**	**ICD Codes**
Heart failure	I50, I43, I099, I110, I130,I132, I255,I420,I425, I426,I427,I428, I429, P290
NSTEMI	I214
Atrial fibrillation	I48
STEMI	I210, I211, I212, I213
Unstable angina	I200
Atrioventricular block	I440, I441, I442, I443
Cardiac arrest	I46
Ventricular tachycardia	I472
Supraventricular tachycardia	I471
Pulmonary embolism	I26, I27.82 (except for I26.01 and I26.90)
Aortic stenosis	I350, I352, I060, I062
Mitral valve prolapse	I340, I341, I051, I052, I058
Pulmonary hypertension	I27.0, I27.2
Hypertrophic cardiomyopathy	I421, I422
Mitral valve stenosis	I342, I050, I052

Supplementary Table 6: Sample sizes in overall data and in the experimental splits.

	Full Data	Development set	Holdout set	First ECG per episode in holdout set
For models with ECG only or ECG, age, sex features				
ECGs (n)	1,605,268	964,741	640,527	297,773
Patients (n)	244,077	146,446	97,631	97,631
Episodes (n)	748,773	451,000	297,773	297,773

ECG: electrocardiogram; N: number.

Supplementary Table 7: ICD and CCI Codes used for identifying the presence of pacemakers.

Procedure	ICD Code	Canadian Classification of Health Interventions (CCI)
Implantable cardioverter-defibrillators implantation	37.94 (ICD-9)	1.HZ.53.GR-FS; 1.HZ.53.HA-FS; 1.HZ.53.LA-FS
Pacemaker	37.78 (ICD-9)	1.HB.53.LA-JA,<br>1.HD.53.GR-JA,<br>1.HZ.37.JA-NN;<br>1.HZ.53.GR-NM; 1.HZ.53.LA-NM; 1.HZ.53.QA-NM; 1.HZ.53.GR-NK; 1.HZ.53.LA-NK; 1.HZ.53.QA-NK; 1.HZ.53.GR-NL; 1.HZ.53.LA-NL; 1.HZ.53.QA-NL; 1.HZ.53.GR-NN; 1.HZ.53.LA-NN; 1.HZ.53.GR-FR; 1.HZ.53.LA-FR
Ventricular assist devices	37.65 (ICD-9)	1.HP.53.LA-QP
Presence of cardiac pacemaker	Z950 (ICD-10)	
Mechanical complication of cardiac electronic device	T821 (ICD-10)	
Encounter for adjustment and management of cardiac device	Z450 (ICD-10)	

Supplementary Table 8: Full forms of ECG measurement names.

Variable	Definition	Unit	Short version
Atrialrate	Atrial rate	Bpm: beats per minute	Atrial Rate
Pdur	P wave duration	Milliseconds	P duration
RRint	RR interval	Milliseconds	RR Interval
Qonset	Q wave onset		Q onset
QTcf	Fridericia rate-corrected QT interval	Milliseconds	Fridericia QTc
Heartrate	Heart rate	Milliseconds	HR (or alternatively, Heart Rate)
PRint	PR interval	Milliseconds	PR interval
QRSdur	QRS duration	Milliseconds	QRS duration
QTint	QT interval	Milliseconds	QT interval
QTcb	Bazett's rate-corrected QT interval	Milliseconds	Bazett's QTc
Pfrontaxis	Frontal P axis	Degrees	Frontal P
i40frontaxis	Frontal QRS axis in initial 40 ms	Degrees	Frontal i40msQRS
t40frontaxis	Frontal QRS axis in Terminal 40 ms	Degrees	Frontal t40msQRS
Qrsfrontaxis	Frontal QRS axis	Degrees	Frontal QRS

Stfrontaxis	Frontal ST wave axis	Degrees	Frontal ST
Tfrontaxis	Frontal T axis	Degrees	Frontal T
Phorizaxis	Horizontal P axis	Degrees	Horizontal P
i40horizaxis	Horizontal QRS axis in initial 40 ms	Degrees	Horizontal i40msQRS
t40horizaxis	Horizontal QRS axis in terminal 40 ms	Degrees	Horizontal t40msQRS
Qrshorizaxis	Horizontal QRS axis	Degrees	Horizontal QRS
Sthorizaxis	Horizontal ST wave axis	Degrees	Horizontal ST
Thorizaxis	Horizontal T axis	Degrees	Horizontal T
tonset	T wave onset		T onset

Supplementary Table 9: Full forms of cardiovascular conditions.

**Conditions**	**Short form**
Atrial fibrillation	AF
Supraventricular tachycardia	SVT
Non-ST-elevation myocardial infarction	NSTEMI
ST-elevation myocardial infarction	STEMI
Pulmonary embolism	PTE
Atrioventricular block	AVB
Hypertrophic cardiomyopathy	HCM
Aortic stenosis	AS
Cardiac arrest	CA
Mitral valve prolapse	MVP
Mitral valve stenosis	MS
Pulmonary hypertension	PHTN
Heart failure	HF
Unstable angina	UA
Ventricular tachycardia	VT
